# Development of Localized Pulmonary Interstitial Emphysema in a Late Preterm Infant without Mechanical Ventilation

**DOI:** 10.1155/2014/429797

**Published:** 2014-03-11

**Authors:** Pritish Bawa, Kultida Soontarapornchai, Agnes Perenyi, Rachelle Goldfisher, John Amodio

**Affiliations:** ^1^Department of Radiology, SUNY Downstate Medical Center, 450 Clarkson Avenue, Brooklyn, NY 11203, USA; ^2^Department of Pediatrics, SUNY Downstate Medical Center, 450 Clarkson Avenue, Brooklyn, NY 11203, USA

## Abstract

Pulmonary interstitial emphysema (PIE) is not an uncommon finding in premature infants with respiratory distress who need respiratory support by mechanical ventilation. PIE has been reported in a few cases of neonates in whom either no treatment other than room air was given or they were given continuous positive end-expiratory pressure (CPAP) support. We present a case of a premature neonate who presented with respiratory distress, in whom PIE and spontaneous pneumothorax (PTX) developed while on CPAP therapy only. The patient was treated conservatively with subsequent resolution of the radiological findings and clinical improvement. No surgical intervention was required. It is important to know that PIE may develop independently of mechanical ventilation. We would like to add this case to the literature and describe the pertinent plain film and computed tomography (CT) findings of this entity, the possible mechanism of development, and the differential diagnosis. A review of the literature is also provided.

## 1. Introduction

Pulmonary interstitial emphysema (PIE) is a form of air leak syndrome. It occurs when the air escapes from the small airway or alveolus into the pulmonary interstitium. It dissects along the bronchovascular bundles and radiates outwards to the periphery of the lung to form subpleural blebs. These blebs may burst through visceral pleura into the pleural space resulting in pneumothorax (PTX). The interstitial air can also pass through the lung hilus directly into mediastinum and cause pneumomediastinum. Further grave complications of interstitial air can be pneumopericardium, pneumoperitoneum, subcutaneous emphysema, and terminally massive air embolism [1].

When infants are given positive pressure ventilation, especially but not exclusively by intubation and mechanical ventilation, a continuous pressure ensures passage of air throughout the interstitial lymphatics. The incidence of PIE is high in ventilated low-birth-weight infants, reported as high as 33% in this group by Greenough and others [2–4]. PIE is more common in premature infants with surfactant deficiency treated with respiratory support in the form of mechanical ventilation.

The incidence of spontaneous air leak syndromes in premature infants is about 1-2%; many infants are asymptomatic. There are few cases of spontaneous PIE in patients on continuous positive airway pressure (CPAP) [4–7].

We wish to add an additional report of a late preterm infant who developed localized pulmonary interstitial emphysema within the right lung, in association with PTX and pneumomediastinum, while on CPAP therapy. We also review the literature on this topic.

## 2. Case Report

A 36-week and 5-day gestation male infant was born to a mother 30 years of age via Cesarean section (C/S) due to repeated C/S. The mother's pregnancy was complicated by preeclampsia. All of her serology was negative, while inadequate treatment for positive GBS, without premature rupture of membranes, was reported. The infant's Apgar scores were 8 and 8 at 1 and 5 minutes, respectively. After 5 minutes of life, he developed nasal flaring, grunting, and subcostal retractions. He was placed on nasal CPAP, with oxygen saturation of 30% and PEEP of 5.

**Figure 1:**
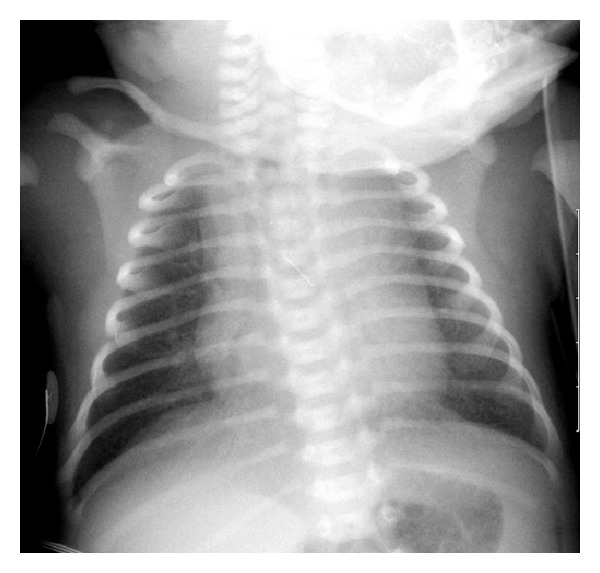
Frontal view of the chest demonstrates mild hyperinflation and mild diffuse air-space disease.

**Figure 2:**
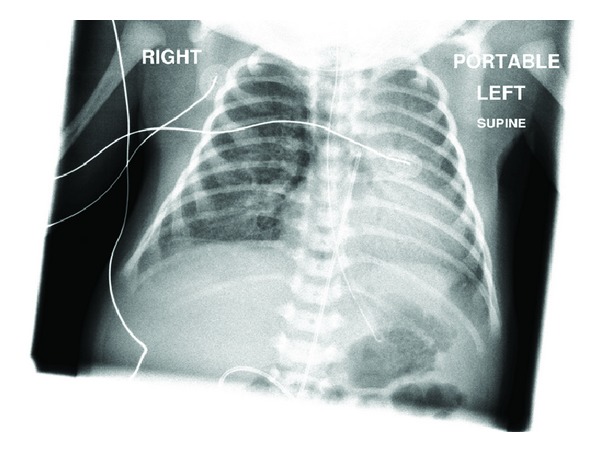
Frontal view of the chest demonstrates the development of multiple air-containing structures within the right lung and pneumomediastinum.

**Figure 3:**
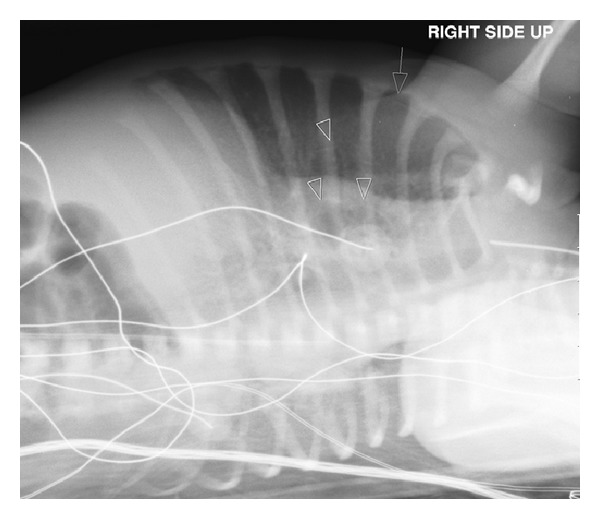
Left lateral decubitus view shows small pneumothorax (arrow) and enlargement of air-containing structures within the right lung (arrowhead).

**Figure 4:**
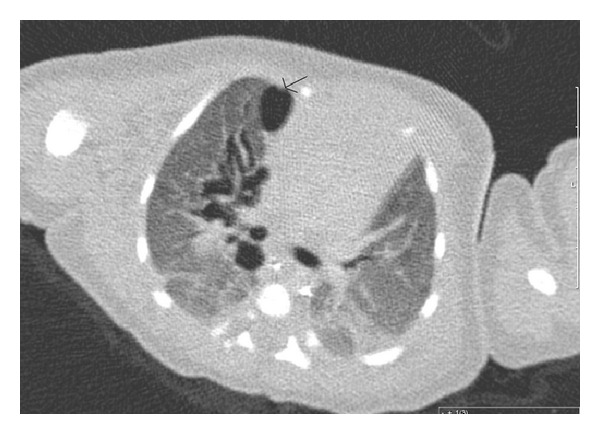
Representative slice from CT scan shows loculated pneumomediastinum (arrow) and multiple branching lucencies in a perivascular distribution, compatible with PIE.

Initial chest X-ray (CXR) (Figure 1) demonstrated diffuse airspace disease and mild hyperinflation. The infant subsequently developed tachypnea and dyspnea, with subcostal, intercostal, and substernal retractions, and required higher oxygen saturation of 40%–45% to maintain oxygen saturation above 95%. A sepsis workup was negative. CXR, at that time, showed the development of multiple air- containing structures within the right lung and pneumomediastinum (Figure 2). A small pneumothorax was seen on a left side down decubitus view. The patient remained stable on CPAP, but subsequent X-rays of the chest demonstrated the development of multiple air-containing cavity-like lesions involving only the right lung and a persistent small pneumothorax (Figure 3). As the patient had not received mechanical ventilation, the diagnosis of a congenital pulmonary airway malformation (CPAM) was considered. A CT scan of the chest at that time revealed multiple linear collections of air following a perivascular distribution, diagnostic of PIE, and loculated pneumomediastinum (Figure 4). The patient remained in stable condition and he was placed in room air; the PIE resolved spontaneously while the patient was in room air. The initial airspace disease was thought to be secondary to retained fetal lung fluid.

## 3. Discussion

PIE and its pathophysiology, both in children and adults, were first described by M. T. Macklin and C. C. Macklin in 1944 [8]. Risk factors for air leak syndromes include prematurity, surfactant deficiency syndrome, meconium aspiration syndrome, amniotic fluid aspiration, infection, low Apgar score, or the need for positive pressure ventilation (PPV) during resuscitation at birth and use of high peak airway pressures on mechanical ventilation.

Preterm infants are at an increased risk for PIE because the perivascular connective tissue is more abundant and less dissectible in preterm than older infants. This allows air trapping in the perivascular space [9, 10].

Lungs with PIE are stiff and the interstitial air can compress adjacent functional lung tissue and vascular structures hampering proper ventilation and pulmonary blood flow. Thus, early detection of this entity is very important to prevent further damage by ventilation under pressure. This sequence of events may be rapid in infants more severely afflicted with surfactant deficiency as well as in those who need high ventilatory pressure for adequate oxygenation.

Findings from animal studies have suggested that PIE may be more associated with structural and maturational factors rather than with overdistention alone [11]. This can explain the occurrence of this entity in premature infants without history of mechanical ventilation.

Very low birth weight (≤1500 grams) is an independent risk factor [12]. In those premature infants, PIE can occur at low mean airway pressure and probably reflects the underdeveloped lung's increased sensitivity to stretch. In a retrospective case-controlled study, 11 (24%) of 45 extremely low-birth-weight (≤1,000 grams) infants developed pulmonary interstitial emphysema [13]. All infants included in the study were treated with a conventional ventilator before the onset of pulmonary interstitial emphysema.

PIE has been described as local or diffuse and acute or persistent [14]. Persistent cases of PIE are categorized pathologically by the presence of giant cells that are seen after ten days of disease [5, 14]. The localized form generally has larger cysts in one or two lobes, whereas the diffuse form commonly has smaller cysts in all the lobes. Cysts in localized PIE may enlarge and result in compression and atelectasis of the adjacent lung.

A number of adverse side effects and complications of CPAP have been described [10]. It has been noted that air leaks, such as pneumothorax and pneumomediastinum, do occur on CPAP [10]. The mechanism may be related to overdistention of the more compliant areas of the lung, with subsequent dissection of air into the mediastinum or pleura.

There have been only a few cases reported for PIE developing in unventilated neonates, like the one we are reporting. PIE in these cases occurred spontaneously and more commonly with CPAP therapy [4, 5].

Freysdottir et al. [15] documented a case of spontaneous PIE in a premature infant born by normal vaginal delivery who was never ventilated. The patient had a complicated course in hospital and subsequently underwent surgery for PIE. The authors proposed another mechanism for the spontaneous development of PIE, namely, laryngomalacia, leading to airway obstruction and subsequent air trapping.

Gurakan et al. [7] described a case of localized persistent PIE in an unventilated very-low-birth-weight premature infant who was on nasal CPAP for 5 days. The infant underwent a CT scan of the chest, which demonstrated multiple thick walled cystic appearing lesions in one lung; since the process was localized to one lung and the patient never received ventilatory support, the diagnosis of a congenital pulmonary airway malformation (CPAM) was considered. However, the process regressed with conservative management.

Bas et al. [9] described a male infant born via spontaneous vaginal delivery at 33 weeks of gestation with a birth weight of 2440 grams. The baby was given CPAP for mild respiratory distress and later developed localized persistent PIE, with slow recovery on conservative management.

Al-Abdi and Singhal [4] described PIE in a premature very-low-birth-weight infant delivered by emergency Cesarean section, who was placed on CPAP. The authors proposed that pregnancy induced hypertension (PIH), hemolysis, elevated liver enzymes, and low platelet count syndrome (HELLP) may be risk factors for PIE. It is interesting that in the case we report there was a history of maternal pregnancy induced hypertension.

Interestingly, a case of spontaneous development of pulmonary interstitial emphysema with PTX and pneumomediastinum complicating pneumonia in a 6-week-old infant was reported by Lee and Im [16]. The patient had no history of resuscitation, nasal CPAP support, or mechanical ventilation. However, this case was associated with lung infection, which may have weakened the structural support of the lung tissue, allowing PIE to develop more readily. There are a few other case reports of PIE in unventilated infants caused by infection/presumed infection. Four of these cases are reported by Crosswell and Stewart [3], Prusnani et al. [17], O'Donovan et al. [18], and Boisset [19].

We are not certain why only the right lung was involved in our case, but we postulate that whatever pressure effects developed as a result of CPAP may have preferentially affected the right lung due to the more vertical course of the right bronchial anatomy. Gaylord et al. [20] observed PIE occurring bilaterally and of those with unilateral disease, 90% had PIE on the right.

Management of PIE includes lateral decubitus positioning, selective bronchial intubation or occlusion, intratracheal surfactant administration, and corticosteroids for the benign disease. Tube thoracostomy has also been used. For the more aggressive or persistent form of the disease high frequency oscillation ventilation, extracorporeal membrane oxygenation, pleurotomy, lobectomy, and pneumonectomy may be used. Surgery is usually required for lesions causing pulmonary dysfunction and/or cardiovascular compromise, which do not resolve with more conservative measures. In the case we are reporting, no special maneuvers were necessary, as the PIE rapidly resolved.

The differential diagnosis of an air-containing lesion in a neonate includes congenital and acquired causes, including CPAM, bronchogenic cyst, congenital lobar emphysema, diaphragmatic hernia, lymphangiectasia, cystic lymphangioma, and sequelae to infection. In the case presented the first radiograph did not reveal any air-containing lesions, but there was a rapid development of localized air- containing structures within the right lung. Since the patient was not on mechanical ventilation, the possibility of CPAM was considered. The CT scan was very helpful in distinguishing PIE from other lesions, as it clearly demonstrated air extending along the peribronchovascular bundles.

**Figure 5:**
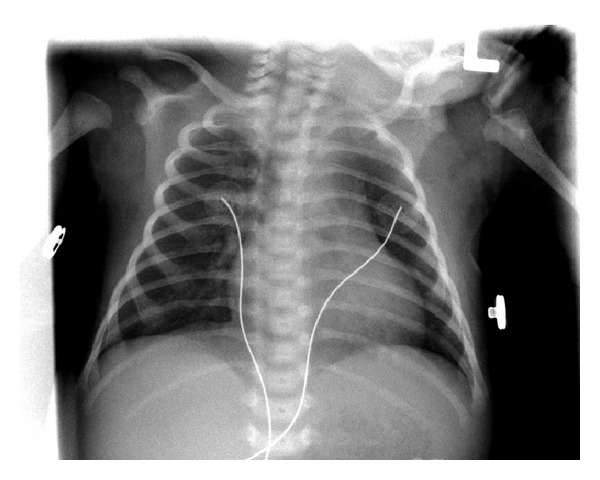
Frontal view of the chest shows complete resolution of PIE, pneumothorax, and pneumomediastinum.

In summary, we present a case of a full-term infant who was placed on nasal CPAP and developed PIE. This case was also complicated by the development of a small PTX and pneumomediastinum. The PIE, PTX, and pneumomediastinum resolved subsequently while the patient was in room air (Figure 5). One should be aware that PIE may develop spontaneously or with nasal CPAP and is not necessarily a complication of mechanical ventilation. CT scans of the chest may help distinguish PIE from other lesions which have a similar appearance on chest X-ray. Conservative management in these cases may lead to complete resolution.
